# Detection and analysis of CpG sites with multimodal DNA methylation level distributions and their relationships with SNPs

**DOI:** 10.1186/s12919-018-0141-x

**Published:** 2018-09-17

**Authors:** Ke Hu, Jing Li

**Affiliations:** Department of Electrical Engineering, Computer Science Case Western Reserve University, 10900 Euclid Ave, Cleveland, OH 44106 USA

## Abstract

DNA methylation levels at cytosine-phosphate-guanine (CpG) sites with multimodal distributions among different samples have been reported recently. One possible explanation for such variability is that genetic variants might affect epigenetic variation. One obvious case is that mutations such as single-nucleotide polymorphisms (SNPs) interrupt CpG sites, resulting in different DNA methylation levels for different genotypes. However, the relationship between genetic variations and epigenetic differences has not been studied thoroughly, partially because of the lack of powerful and robust methods to survey genome-wide CpG sites with multimodal methylation level distributions (mmCpGs). In this article, we develop a Gaussian mixture-model clustering (GMMC)–based approach to systematically detect all mmCpGs across the genome based on the GAW20 data set. In total, 3785 and 3847 mmCpGs have been identified in pre- and posttreatment data sets, respectively. Result analysis shows that approximately 68 to 70% of mmCpGs detected from unrelated individuals either have direct overlaps with SNPs or have associations with nearby SNPs, suggesting a strong correlation between SNPs and mmCpGs. Comparison with an existing approach illustrates that our GMMC-based method is more consistent when the number of samples decreases. In conclusion, mmCpGs may reveal important connections between genetics and epigenetics and they should be carefully identified and evaluated.

## Background

DNA methylation is one of the most widely used epigenetic marks and plays an important role in gene regulations, which may result in phenotypic differences among different individuals, as well as phenotypic differences of the same individual, before and after treatments [1]. Although epigenetics is traditionally defined as heritable changes in gene activities that do not involve genetic mutations, recent studies suggest associations exist between genetic variants and differences in DNA methylation levels [2, 3]. Large-scale genome-wide DNA methylation profiling (e.g., using Illumina Infinium Human Methylation450 Beadchip, aka Illumina 450 K), together with genome-wide genotyping assays using single-nucleotide polymorphism (SNP) arrays, enables studies of associations between genetic variations and differences in DNA methylation levels.

Even though many studies have treated DNA methylation levels as a quantitative trait and performed so-called methylation quantitative trait locus analysis, two recent studies [4, 5] have investigated multimodal distributions of methylation levels at cytosine-phosphate-guanine (CpG) sites, primarily as a quality control step to correct methylation signals from Illumina 450 K chips. Daca-Roszak et al. [4] studied relationships between SNP genotypes and methylation levels of 96 CpG sites from European and Asian populations. They observed multimodal distributions among individual samples for CpG sites with SNPs. However, their study was limited to a very small subset of CpG sites and only considered CpG sites that physically overlapped with SNPs. In another attempt, Andrews et al. developed an interval-based clustering method called *Gaphunter* to identify CpG sites with multimodal distributions (mmCpGs) [5], which was implemented in the Bioconductor package *minfi* [6]. Gaphunter first sorts individual DNA methylation levels of candidate CpG sites and then groups them into clusters with predefined methylation-level thresholds. An optional post-processing step can be used to exclude outlier-driven clusters, which are defined as clusters with smaller sizes relative to the total sample size and the size of the largest cluster. This simple algorithm is fast and works well for moderate-size data sets with little experimental measurement noise of methylation levels. The authors also explored applications of mmCpGs such as probe quality control and population stratification adjustment. However, threshold-based approaches such as Gaphunter are sensitive to noise levels and sample size.

To overcome those limitations, we propose a more generic and more robust clustering method to identify mmCpGs. The method is based on a Gaussian mixture model and we apply it to the GAW20 data sets to identify mmCpGs. We further check the relationships between SNPs and mmCpGs in terms of direct overlaps of their genomic locations, as well as statistical associations between mmCpG clusters and genotypes of SNPs that are physically close to mmCpGs. Analysis result shows that approximately 68 to 70% of mmCpG sites are associated with some SNPs within their 100 kbp neighborhood, suggesting high concordances between mmCpG clusters and individual genotypes. In comparison with Gaphunter, results show that our approach is more robust and more stable than that of Gaphunter.

## Methods

### Data

In this study, we analyzed the genome-wide DNA methylation data before treatment and after treatment, as well as dense SNP genotype data provided by GAW20. There are 995 individuals from 182 families in the pretreatment methylation data set and 530 individuals from 153 families in the posttreatment methylation data set. Among the 1525 individuals, 823 have been genotyped. Of the individuals in the pretreatment dataset, 717 have both methylation and SNP data; in the posttreatment data set, 507 individuals have both methylation and SNP data. We performed mmCpG predictions on all individuals with methylation data, separately for the pretreatment and posttreatment data sets. Because of time limitations, we randomly picked 1 member from each family to assess associations between mmCpGs and genotypes. Association between mmCpGs and SNPs in related individuals will be examined in future studies. The number of CpG sites included is 463,995. The DNA methylation level of each CpG site in an individual is a numeric value between 0 and 1. The SNP array data consists of 718,566 SNPs. The genotype data are defined as 0, 1, or 2, representing the number of copies of the coded allele. Because genotype and DNA methylation data may contain missing value for some SNPs or CpG sites, we only include those individuals who have both genotypes and DNA methylation information when associating cluster labels with genotypes.

### Gaussian mixture-model clustering

The goal of our method, Gaussian mixture-model clustering (GMMC), is to identify clusters of individuals that have distinct distributions of DNA methylation levels for each CpG site without using any prior knowledge of genotypes or phenotypes. Gaussian mixture model (GMM) is one of the most widely used model-based clustering algorithms that is suitable for identifying cluster structures from a mixture of multiple distributions. A GMM is a weighted sum of *M* component Gaussian densities as shown in formula below,

1 $$ p\left(x|\lambda \right)={\sum}_{i=1}^M{w}_ig\left(x|{\mu}_i,{\sigma}_i^2\right) $$

where *w*_*i*_ is the weight of component *i*, and

$$ g\left(x|{\mu}_i{\sigma}_i^2\right),i=1,\cdots, M $$

are the component Gaussian densities.

The assumption is that when methylation levels are affected by genotypes, each distinct genotype corresponds to a different distribution. In a population with different types of genotypes, their methylation levels will exhibit the characteristics of a mixture distribution. In our study, we use the *mixtools* [7] to perform GMMC, which will estimate model parameters for each cluster using the Expectation-Maximization algorithm for a given number of clusters. It also provides posterior probabilities of a sample belonging to each of the clusters. An evaluation metric, such as Bayesian information criterion (BIC) and log-likelihood of the mixture-model, can be used for model selection.

### Detection of multimodal CpG sites

To determine the best number of clusters, we try different numbers of clusters iteratively and determine the best model using the BIC criteria. More specifically, we apply the following algorithm:

0. Starting with k = 1, calculate BIC_1_ based on unimodal GMM_1_. BEST_MODEL = GMM_1_.

1. If k > MAX_K, stop iteration.

Otherwise, k = k + 1. Apply GMMC with given k components. Obtain BIC_k_ and GMM_k_.

2. If BIC_k_ > BIC_k − 1_ + BIC_INC_THRESHOLD, BEST_MODEL = GMM_k_. Continue to step 1.

Otherwise, stop iteration.

Given the property of our specific application, we set the *MAX_K* as 3, corresponding to the 3 distinct genotypes of some SNPs that are potentially associated with the mmCpG. The BIC incremental threshold (*BIC_INC_THRESHOLD*) is used to control the model complexity. A larger number of clusters are meaningful only if it’s the larger number’s BIC is substantially higher than the BIC with a smaller number of clusters. In practice, a higher value of the threshold will allow the method to be less sensitive. We set *BIC_INC_THRESHOLD = 100* in our analysis so that our results are more conservative.

Once the model is fixed, each individual is assigned to the cluster for which the posterior probability is highest. Our method also incorporates a postprocessing step that uses several thresholds to filter out low-quality clusters. First, the largest cluster cannot be too big. If the fraction of the largest cluster is greater than *1 − OUT_CUTT*, where *OUT_CUTT* is a user-specified parameter, the mmCpG will be excluded from further analysis. Second, samples within each cluster should have small variance, controlled by a threshold *MAX_STD* for the maximum allowed standard deviation in each cluster. Finally, we require that cluster centers should be separable from each other, which is controlled using a threshold *MIN_MEAN_DIFF*. In our study, we set *MAX_STD = 0.1* and *MIN_MEAN_DIFF = 0.2*.

### Associating GMM cluster labels with genotypes

To study the relationships between genotypes and mmCpGs, we evaluated genotype data and GMM cluster labels together to assess the strength of associations. In our study, we included all SNPs located less than 50 kb on either side of an mmCpG site. For each pair of a SNP and an mmCpG, we first constructed the contingency table for 3 genotypes and 3 cluster labels. Then a chi-square *p* value was calculated and corrected by Bonferroni correction for multiple testing. Among all the nearby SNPs around an mmCpG, only the SNP with the minimum *p* value is considered as the measure of SNP-mmCpG association. Finally, a critical value of 0.001 (after Bonferroni correction for multiple testing) was used to determine if an mmCpG has strong association with at least 1 nearby SNP.

## Results

### Genome-wide survey of mmCpG sites in the GAW20 data set

**Fig. 1:**
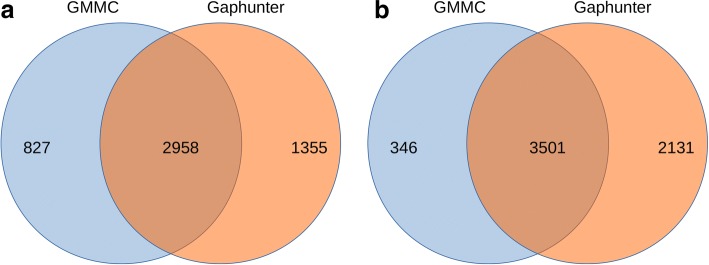
Venn diagram of GMMC and Gaphunter results on the pretreatment (**a**) and posttreatment (**b**) data set

**Fig. 2:**
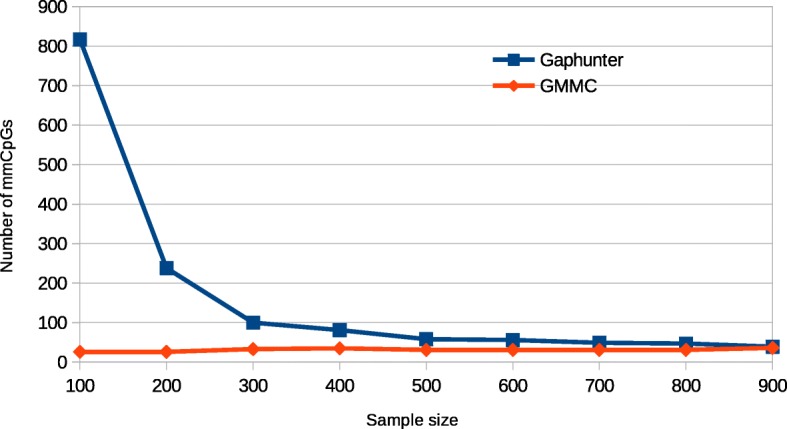
Number of mmCpGs detected by GMMC and Gaphunter in different sample sizes

We applied our GMMC-based method on both pretreatment (995 individuals) and posttreatment (530) Illumina 450 K data sets and detected 3785 and 3847 mmCpGs, respectively. A significant majority of them (2965 mmCpGs) were found in both data sets. Of the mmCpGs, 820 and 882 were found unique in pre- and posttreatment data sets, respectively. To compare our method with Gaphunter, we also applied Gaphunter on the same data sets. Gaphunter identified 4313 and 5632 mmCpGs in pre−/posttreatment data sets, respectively. Approximately 78% (pretreatment) and 91% (posttreatment) of mmCpGs identified by our method were also included in Gaphunter result. Moreover, the number of mmCpGs identified by our method alone is much smaller than the number of mmCpGs identified by Gaphunter alone, which indicates that our method is much more conservative than Gaphunter (Fig. 1). To evaluate the sensitivity to sample size of both methods, we randomly picked different numbers of individuals from all individuals in the pretreatment DNA methylation data and applied both methods on chromosome 21 of the sub-data sets. Our analysis shows that Gaphunter has many more mmCpGs with small sample sizes and the number of mmCpGs identified decreases as the sample size increases (Fig. 2). In contrast, our method is very stable for all tested sample sizes. Many of the reported mmCpGs by Gaphunter when using small sample sizes are likely false positives. In summary, results indicate that our method is more conservative with small sample sizes and more stable than Gaphunter.

### Association between mmCpGs and SNPs

**Fig. 3:**
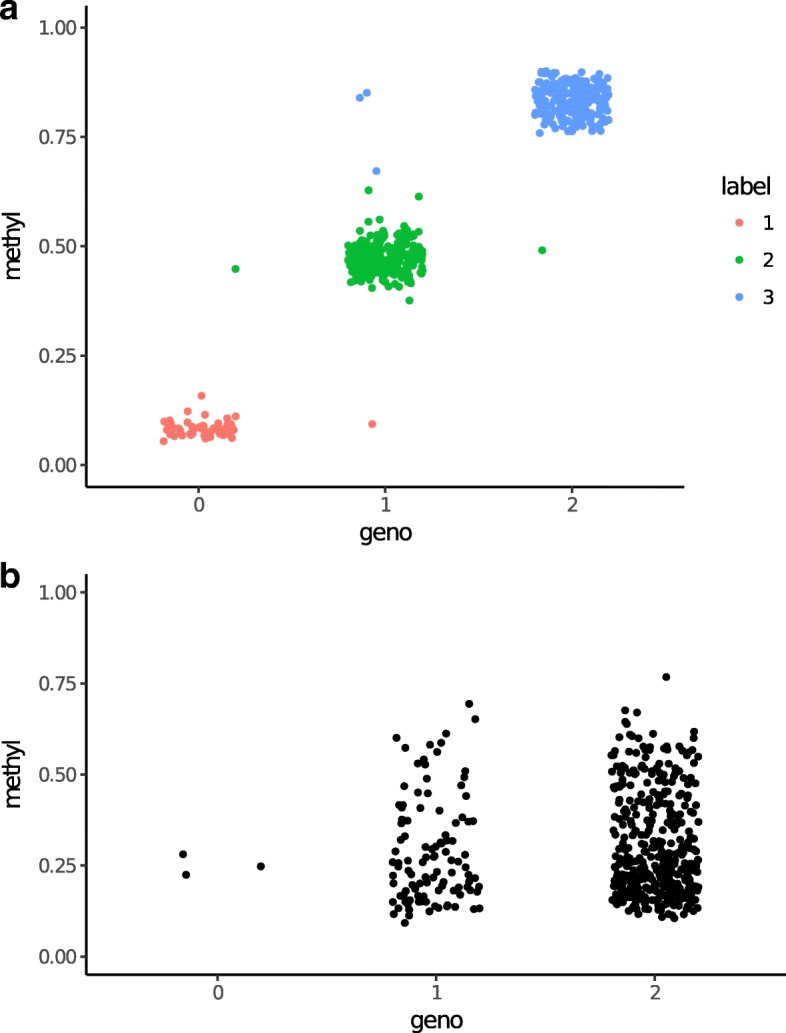
**a** an example of an mmCpG with a genotyped SNP physically overlapped with its location. Each point represents an individual. **b** an example of a non-mmCpG with a genotyped SNP physically overlapped with its location. Distributions of different genotype groups are similar

To investigate the relationships between mmCpGs and SNPs, we separate mmCpGs into two categories: (a) mmCpGs that have a SNP directly overlapping with it (either at C position or G position); and (b) mmCpGs with no directly overlapped SNPs, but which have strong associations with some SNPs in close physical proximity. Because family structure may have impact on correlation between genotype and methylation level, we further conducted analysis on unrelated individuals (182 pretreatment/153 posttreatment), which detected 3014 and 3128 mmCpGs in pre- and posttreatment data sets, respectively. There are in total 453 CpG sites directly overlapping a SNP; 180 of which are detected as mmCpGs in pretreatment datasets and 190 are detected in posttreatment data sets. Figure 3 shows examples of an mmCpG with overlapped SNPs and a non-mmCpG site with overlapped SNPs.

**Fig. 4:**
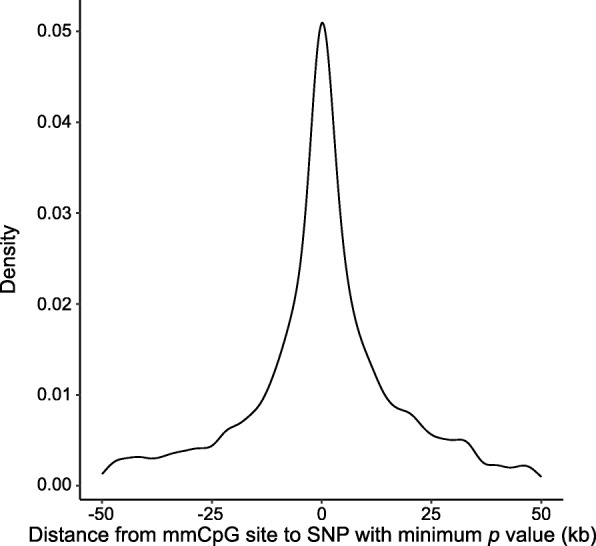
Distance distribution from mmCpG sites to their associated SNPs

In addition to direct overlaps at their locations, nearby SNPs may affect/interact with mmCpGs. We examined SNPs located within 50 kb on either side of each CpG site (100-kb window). By matching genotypes and GMM cluster labels, we measured the association between mmCpGs and their nearby SNPs based on a contingency table. Results show that approximately 68 to 70% of mmCpGs in both pre- and posttreatment data sets are associated with at least one of the nearby SNPs (Table 1). This observation supports our hypothesis that most of mmCpGs are somehow affected by SNPs. Moreover, we found that the most associated SNPs were located within 20 kb of the mmCpGs (Fig. 4).

**Table 1:** Summary of mmCpGs pre- and posttreatment

	mmCpG_pre	%	mmCpG_post	%
All result	3014	–	3128	–
*p* ≤ 0.001	2073	68.78	2207	70.56
*p* > 0.001	941	31.22	921	29.44

## Discussion

We have proposed a novel GMMC-based method to detect genome-wide mmCpGs generated from Illumina 450 K chips. We applied this method on the GAW20 data set and found that the majority of mmCpGs are associated with SNPs that are either directly overlapping with CpG sites or are in close proximity to CpG sites. Empirical analysis demonstrates that our method is more stable than Gaphunter, a thresholding-based method. The ideas that underlie threshold-based clustering and model-based clustering are quite different. Threshold-based methods such as Gaphunter use a fixed cutoff value to draw a boundary to separate data points, which may not be able to capture characteristics of different clusters. First, the choice of cutoff values is mostly arbitrary. The same cutoff values may not be valid in different data sets or, even worse, they may not be valid for different CpG sites in the same data set, because methylation level distributions of different CpG sites may have different characteristics, that is, some CpG sites have larger gaps between clusters than other CpG sites. Moreover, the cutoff values can be quite sensitive to sample sizes. When the sample size is small, the distribution of DNA methylation levels among individuals will be sparse and the within-cluster distances may be big. Threshold-based methods are prone to false positives. When the sample size is large, the distribution is dense and clusters may have overlaps, making it hard for threshold-based approaches to correctly cluster samples. Unlike threshold-based methods, model-based clustering methods, including our proposed GMMC method, are designed to obtain models that fit the distributions and can naturally capture the characteristics of cluster structures. Consequently, model-based clustering methods usually provide more accurate results. In addition, the GMM can detect clusters with identifiable overlaps.

The current study mainly focused on detection of mmCpGs. Our findings suggest that there might be some connections between genetics and epigenetics. One should not treat mmCpGs as irregularities and filter them out from further analysis. Instead, careful characterization after identification is needed to better understand the biological significance of mmCpGs. In the future, we will investigate the association between mmCpGs and genotypes and explore how these mmCpGs might be related to phenotypes.

## Conclusions

A Gaussian mixture-model clustering algorithm was developed and applied on the GAW20 data set to detect CpG sites with multimodual methylation levels. The result of our analysis shows that a large number of detected mmCpGs either directly overlap with SNPs or have strong associations with nearby SNPs, suggesting correlations between genetic mutations and methylation level variations.
